# Molecular analysis of acetylcholinesterase gene in field-collected populations of *Musca domestica* (Diptera: Muscidae) in Northwestern Iran

**DOI:** 10.1093/jisesa/iead054

**Published:** 2023-07-22

**Authors:** Delnia Adib, Abbas Jafari, Elena Silivanova, Hamidreza Basseri, Saber Gholizadeh

**Affiliations:** Health and Biomedical Informatics Research Center, Urmia University of Medical Sciences, Urmia, Iran; Medical Entomology Department, School of Public Health, Urmia University of Medical Sciences, Urmia, Iran; Cellular and Molecular Research Center, Cellular and Molecular Medicine Institute, Urmia University of Medical Sciences, Urmia, Iran; Department of Clinical Toxicology, School of Medicine, Urmia University of Medical Sciences, Urmia, Iran; All-Russian Scientific Research Institute of Veterinary Entomology and Arachnology, Branch of Federal State Institution Federal Research Centre Tyumen Scientific Centre, Siberian Branch of the Russian Academy of Sciences (ASRIVEA – Branch of Tyumen Scientific Centre SB RAS), Institutskaya St. 2, Tyumen, 625041, Russian Federation; Vector Biology and Control of Diseases Department, School of Public Health, Tehran University of Medical Sciences, Tehran, Iran; Health and Biomedical Informatics Research Center, Urmia University of Medical Sciences, Urmia, Iran; Medical Entomology Department, School of Public Health, Urmia University of Medical Sciences, Urmia, Iran

**Keywords:** housefly, acetylcholinesterase, polymorphism, novel mutation

## Abstract

Nowadays, pyrethroid (Py) insecticides are commonly used against household insect pests and housefly. The combination of Py and organophosphates (OP) are also utilized to combat these insects. The resistance status of Iranian housefly populations to them and carbamate (CB) insecticides is uncertain. This study investigates the presence of acetylcholinesterase (AChE) mutations related to the resistance of *Musca domestica* to OP and/or CB insecticides in Northwestern Iran. Nucleotides 1041–1776, based on their positions in the ACE gene of aabys strain, were amplified and sequenced in houseflies collected from West Azerbaijan, Gilan, and Ardebil Provinces, Iran. Among 12 single-nucleotide polymorphisms detected, 3 mismatches were found at nucleotides 1174 (T/A, G), 1473 (G/T, C), and 1668 (T/A), leading to amino acid substitutions in V260L, G342A/V, and F407Y positions with various combinations. Genotyping results showed that 85% of specimens had at least one of these substitutions. In addition, the Iranian housefly population was composed of 5 insensitive and sensitive alleles. For the first time, the current study reports the presence of V260L, G342A, G342V, and F407Y substitutions in *M. domestica* specimens collected from Northwestern Iran. The selection of multiple alleles in field populations might be due to the application of various pesticides/insecticides during extended periods in the region. These molecular levels signify the presence of control problems in the area and the need for developing effective control strategies for such populations.

## Introduction

Houseflies have a global distribution and are intimately involved in human activities such as restaurants, hospitals, food centers, fish and food markets, and slaughterhouses (Zafar et al. 2014, Paliy et al. 2018, Issa 2019). This fly species is vital for health, economics, and veterinary purposes and transmits more than 130 human and animal pathogens, mostly bacteria (Wang et al. 2012, Khamesipour et al. 2018); it also causes myiasis in humans (Dogra and Mahajan 2010, Dehghani et al. 2012).

Insecticides, mostly organophosphates (OP) insecticides, have broadly been utilized in many countries to control flies (Scott et al. 2009, Wang et al. 2012, Scott et al. 2014, Zafar et al. 2014). A major feature of OP insecticides is the prevention of the acetylcholinesterase (AChE) enzyme (Devonshire 1975). When the insect is poisoned, the active site of the enzyme is changed and irreversibly inhibited. As a result, acetylcholine accumulates in synapses and causes the permanent opening of acetylcholine receptors, giving rise to the insect's death (Başkurt et al. 2011). The extensive and frequent use of insecticides has resulted in the resistance of disease vectors to these compounds because of short generation time, large population size, and intense selection pressure (Başkurt et al. 2011). Until 14 May 2023, 425 cases of resistant houseflies have been recorded in Arthropod Pesticide Resistance Database from different parts of the world (https://www.pesticideresistance.org/). The emergence of resistant populations is one of the significant problems of insect control (Hemingway and Ranson 2000, Karunamoorthi and Sabesan 2013).

Molecular and genetic studies have reflected that resistance of insects to insecticides is mainly associated with mutations in a limited number of genes (Qiu et al. 2012, Riaz et al. 2022, Roca-Acevedo et al. 2023). In *Musca domestica*, only one ACE, called ACE2, encodes the AChE enzyme and is the molecular basis of insecticide resistance due to the insensitive AChE enzyme (Kozaki et al. 2001b, Walsh et al. 2001, Ffrench-Constant et al. 2004, Guo et al. 2017). Point mutations in the gene encoding the enzyme AChE lead to the production of a modified enzyme, which is the primary mechanism of resistance to pirimiphos-methyl,azinphos-methyl in *M. domestica* and some other insects, including green bugs and potato Colorado beetle (Zhang et al. 1999, Alzabib et al. 2023). Amino acid substitutions in the AChEprotein, V260L, G342A/V, F407Y, and G445A are linked with the resistance to OP insecticides in the housefly, based on a comparison between ACE gene in resistance and susceptible strains of *M. domestica* (Walsh et al. 2001). An association has also been demonstrated between A316S mutation and 5 above-mentioned mutations alone or in combination, conferring houseflies’ resistance to OP/carbamate (CB) insecticides (Qiu et al. 2012). For effective management, identification, and monitoring of insecticide resistance, there is a need for quick, simple, and precise methods such as molecular techniques that have previously been used to identify mutations in housefly (Qiu et al. 2012).

Arthropod chemical control programs have a long history in Iran. During the past decades, organochlorides (OCs), OPs, CBs, and pyrethroids (Pys) have been 4 major classes of insecticides utilized to control houseflies, mosquitoes, and cockroaches, as well as to protect crops (Zaim et al. 2017, Kassiri et al. 2020). Recently, 2 other groups of compounds, namely pyrroles and phenyl pyrazoles (Liu 2015, Kassiri et al. 2020) [https://www.pesticideresistance.org/], have been added to the list of previous compounds. About 37% of annually used pesticides in Iran during 2012–2014 (14,000 tons) belonged to insecticides and acaricides (Zaim et al. 2017). The high-volume use of pesticides is required for food production for the Iranian population, which has been doubled during the last 40 yr (Karimi et al. 2019).

As far as is known, there is limited information on the sequence of ACE alleles in field-collected *M. domestica* populations. Most studies have employed laboratory strains to describe ACE alleles frequency (Kozaki et al. 2001a, 2001b, Walsh et al. 2001, Kozaki et al. 2009). However, a new mutation (A316S) in laboratory strains and only one resistance allele in field-collected strains have been reported (Kozaki et al. 2009). In the current study, ACE alleles were sequenced in the field-collected specimens of houseflies from Northwestern Iran at the nucleotide positions 1041–1776 of aabys strain, with the GenBank accession No. AF281161 (Kozaki et al. 2009). This region includes amino acid positions of 260, 316, 342, and 407, diagnostic points for OP and CB resistance. So far, there has been no exploratory research on the resistance of houseflies to OP insecticides in Iran. Thus, given the role of houseflies in mechanical transmission of diseases to humans, investigating and monitoring insecticide-resistant status of *Musca domestica* using new techniques could be more evident.

## Materials and Methods

### *Musca domestica* Flies

**Fig. 1.**
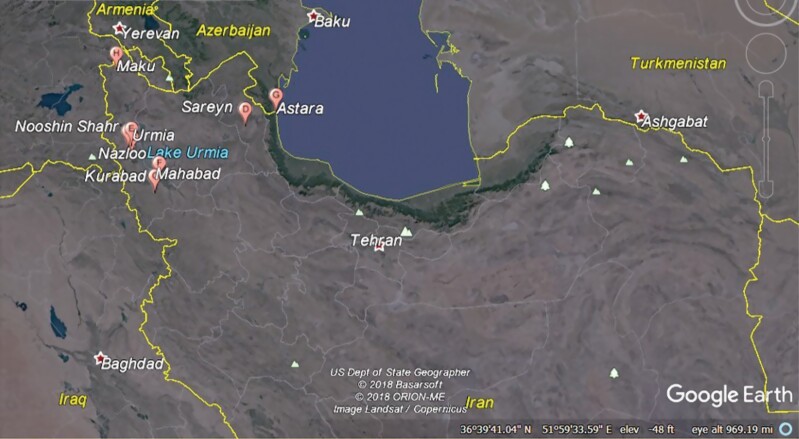
Map of Iran indicating the sample collection locations and their situation in the neighborhood of Azerbaijan, Iraq, and Turkey countries.

Samples of houseflies were collected from West Azerbaijan, Ardebil, and Gilan Provinces of Iran using sweep nets during the year 2015. The details of the study area are summarized in Fig. 1 and Table 1. Samples (in ethanol 70%) were transported to the Medical Entomology Laboratory at Urmia University of Medical Sciences (UMSU) and stored at –20 °C. Morphological keys (Borror and DeLong 1971, Carvalho and Mello-Patiu 2008) were used for taxonomic identification.

**Table 1.** Collection sites for the haplotypes identified in this study

Accession numbers	Province	District	Geographical coordinates
MK257688, MK257697, MK257699	West Azerbaijan	Urmia	45° 2ʹ N, 37° 19ʹ E
MK257685, MK257686, MK257687		Nazloo	44° 58ʹ N, 37° 40ʹ E
MK257691, MK257692, MK257693		Nooshin Shahr	45° 1ʹ N, 37° 43ʹ E
MK257700, MK257701, MK257704		Koorabad	45° 2ʹ N, 37° 19ʹ E
MK257689, MK257690		Mehabad	45° 43ʹ N, 37° 9ʹ E
MK257694		Makou	44° 33ʹ N, 39° 17ʹ E
MK257695, MK257696	Ardebil	Sarein	48° 2ʹ N, 38° 5ʹ E
MK257702, MK257703, MK257704	Guilan	Astara	48° 52ʹ N, 38° 24ʹ E

### DNA Extraction and PCR Amplification

DNA was extracted from houseflies using YTA genomic DNA extraction mini kit (Yekta Tajhiz Azma, Tehran, Iran) based on the manufacturer’s instructions with slight modifications (Kamdar et al. 2019). The DNA was dissolved in 100 µl of ddH_2_O (pH 7.5–9.0) and kept at −20 °C until use in molecular studies. The ACE fragment was amplified by primer pairs S90MdAce (CATCTAAAACCGATCAGGACCATTTAATAC) and AS89MdAce (TCATCTTTAACATTTCCAATCAGAATATCG) (Kozaki et al. 2009). The 25-μl PCR reactions contained 1 μl of genomic DNA, 1 μl of each primer, 9.5 μl of ddH_2_O, and 12.5 μl of Master Mix (Yekta Tajhiz Azma). PCR amplifications were set up in a hot start at 94 °C for 3 min, followed by 40 cycles of denaturation at 95 °C for 30 s, annealing at 55 °C for 30 s, and extension at 72 °C for 90 s, and final elongation step at 72 °C for 10 min (Kozaki et al. 2009). PCR products electrophoresed on 1% agarose gel were stained with safe stain (YTA Cat No. YT0001). Ultimately, 20 PCR products were directly subjected to sequencing using an ABI-377 automatic sequencer (Takapouzist, Tehran, Iran) in both strands.

### Sequence Analysis

The sequences related to Iranian specimens were checked with Chromas software version 2.31 (www.technelysium.com.au/chromas.html) in both directions. The multiple sequences were compared using the Basic Local Alignment Search Tool (BLAST) and aligned using both MEGA6 (Tamura et al. 2013) and Clustal Omega (Sievers and Higgins 2014). Phylogenetic trees were constructed based on 20 nucleotide and amino acid sequences of the ACE gene in houseflies from Iran and 15 sequences extracted from the GenBank (GenBank IDs: FJ174253–FJ174267 as V1–V15 alleles) (Kozaki et al. 2009). To synchronize our sequences with those derived from the GenBank, 148 nucleotides (49 amino acids) from the beginning and 74 nucleotides (24 amino acids) from the end of the Iranian sequences were deleted. Phylogenetic trees were constructed as per the Tamura–Nei (TrN) model of evolution (Tamura and Nei 1993) and analyzed with maximum likelihood algorithms, the most popular approaches to use the likelihood ratio test. Evolutionary analysis was conducted by the aid of MEGA6 (Tamura et al. 2013). Bootstrap replicates inferred from 1,000 replicates are indicated in branches to represent the evolutionary history of the taxa analyzed. Frequencies of RR, RS, and SS genotypes were calculated by dividing the number of houseflies of each genotype by the total number of analyzed houseflies. All the frequencies were scored based on the chromatogram results and the double-peak signal (Brownell et al. 2020). Nucleotide sequences are available in the GenBank, European Molecular Biology Laboratory (EMBL), and DNA Data Bank of Japan (DDBJ) databases (GenBank: MK257685–MK257704).

## Results

A fragment (~800 bp) of the ACE gene was amplified, and sequences in housefly specimens were randomly selected from each study area. Deduced sequences were compared with those of aabys-susceptible strain (Kozaki et al. 2001a) and V1–V15 alleles (Supplementary Fig. 1) (Kozaki et al. 2009).

**Fig. 2.**
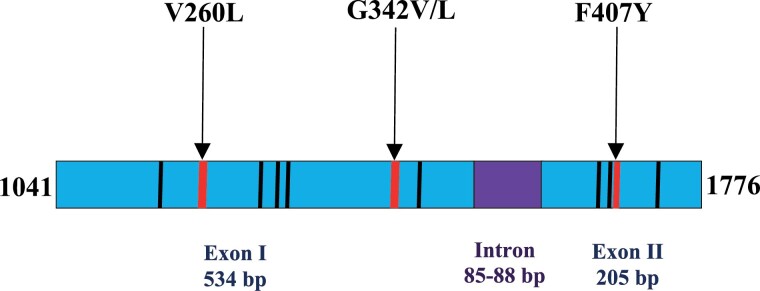
Schematic of the sequence acetylcholinesterase gene in this study. Position of nonsynonymous and synonymous mutations are depicted in red and black lines, respectively.

**Fig. 3.**
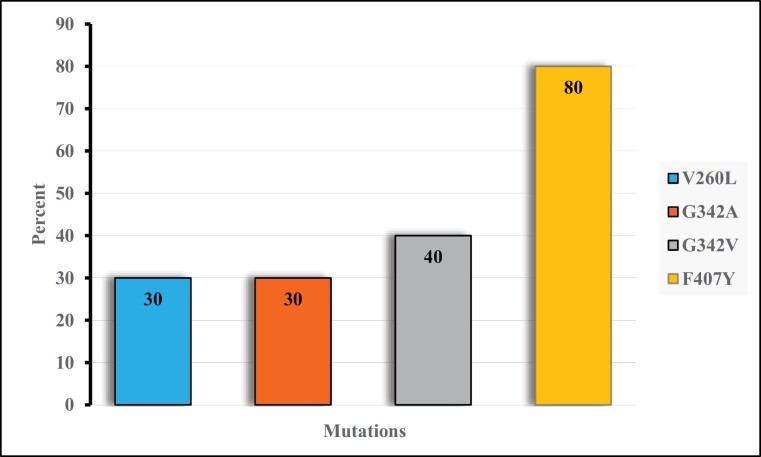
The frequency of resistance mutation among *M. domestica* specimens collected from Iran.

The sequenced fragment of the ACE gene (from nucleotide numbers 1041–1776 of strain aabys AF281161 coding region of 240 amino acid residues mature protein sequence) was composed of an intron (85–88 bp) and 2 flanking exons (534 bp and 205 bp) for a total length of about 824–827 bp (Fig. 2). Regardless of size variation, sequence similarity within Iranian specimens and between V1–V15 alleles (Kozaki et al. 2009) was 98.26–100%. Nucleotide sequence similarity between Iranian sequences and aabys-sensitive strain in both exon regions was 98.65–100%. There were 11 single-nucleotide polymorphisms as a transition (*n* = 7) and transversion (*n* = 4) in exon I (*n* = 7) and exon II (*n* = 4) regions (Fig. 2 and Supplementary Fig. 1). There was also heterozygosity in the ACE gene in positions 1174 (T/A, G) and 1473 (G/T, C). Mismatches in exon I resulted in an amino acid substitution in positions V260L and G342A/V (Fig. 2). Prevalence of the 2 first substitutions was 30%, while the last was 40%. Among 4 mutations found in the exon II region, T/A mutation (1668) was nonsynonymous and led to F407Y substitution in 80% of the sequenced specimens (Figs. 2 and 3).

Amino acid sequences in 16 samples were polymorphic in more than one position, and the GenBank ID: MK257704 was polymorphic only in one position. The remaining 3 samples (GenBank IDs: MK257686, MK257694, and MK257699) had no amino acid substitution and were considered sensitive specimens (Table 2). Therefore, in addition to sensitive alleles, 5 different insensitive alleles were identified in Iranian sequences, as compared to V1–V15 alleles (Table 2). Alleles I, III, and V had V260-V342-Y407, L260-A342-Y407, and V260-A342-Y407 combinations and were similar to V14 (GenBank ID: FJ172466), V15 (GenBank ID: FJ172467), and V10 (GenBank ID: FJ172462), respectively (Kozaki et al. 2009). L260-G342-Y407 and L260-G342-F407 combinations were novel and specific for the Iranian housefly population (Table 2). Allele I, including 7 sequences (Table 2), was the prevalent allele (40%); however, the prevalence of this allele was 10% and 5% for novel alleles, L260-G342-Y407 and L260-G342-F407, respectively. Genotyping based on chromatogram results revealed that only MK257690 sequence was Ace-resistant homozygous mutant (RR) in V260L and G342A positions. The most frequent ACE-resistant heterozygous mutation (RS) was detected in F407Y (80%), G342V, G342A, and V260L, respectively. The detail of genotyping results is depicted in Table 3.

**Table 2.** Frequency of AChE alleles and related amino acid substitutions in sequences of 20 houseflies collected from Iran and their comparison with alleles reported by Kozaki et al. (2009)

Alleles	Accession numbers	Lab. alleles^a^	260	316	342	407	Type^b^
Allele I	MK257685, MK257687, MK257688, MK257689, MK257691, MK257696, MK257697, MK257698	V14	V	A	V	Y	Insensitive
Allele II	MK257695, MK257700	—	L	A	G	Y	Putative insensitive
Allele III	MK257690, MK257692, MK257693	V15	L	A	A	Y	Insensitive
Allele IV	MK257704	—	L	A	G	F	Putative insensitive
Allele V	MK257701, MK257702, MK257703	V10	V	A	A	Y	Insensitive
Allele VI	MK257686, MK257694, MK257699	V1–V9, V12,V13	V	A	G	F	Sensitive

^a^Lab allele VII was not detected in the current study.

^b^AChE that is insensitive or sensitive to inhibition by organophosphate and/or carbamate insecticides.

**Table 3.** Genotyping results of obtained sequenced based on chromatogram results and the double-peak signal

Amino acid	Genotype^a^	
	R/R	S/R
V260L	MK257690	MK257692, MK257693, MK257695, MK257700, MK257704
G342A	MK257690	MK257692, MK257693, MK257701-03
G342V	—	MK257685, MK257687-89, MK257691, MK257696-98
F407Y	—	MK257685, MK257687-93, MK257695-98, MK257700-03

^a^S and R are susceptible and resistant alleles, respectively.

**Fig. 4.**
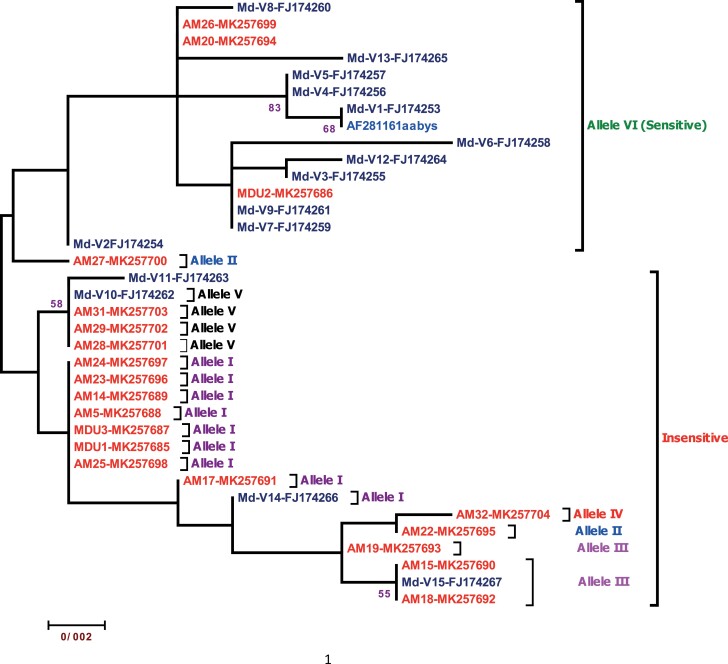
Maximum likelihood tree based on ACE nucleotide sequences for 20 Iranian housefly specimens and 15 GenBank sequences. Bootstrap values (1,000 replicates) > 50 are shown above the lines. AF281161 aabys and V1–V15 (FJ174253–FJ174267) were extracted from the GenBank (Kozaki et al. 2009).

The constructed phylogenetic tree based on amino acid and nucleotide sequences was different in topology (Fig. 4). The variation between the 2 trees was due to synonymous nucleotide mutation(s). For instance, V14 formed a branch with MK257685, MK257687, MK257688, MK257689, MK257691, MK257696, MK257697, and MK257698 in the amino acid phylogenetic tree, whereas in the nucleotide-based phylogenetic tree, V14 was clustered in another branch near MK257691, owing to a variation in nucleotide 1473 (G/T) (Supplementary Fig. 1). The similarity of exon sequences in V10, MK257701, MK257702, and MK257703 with MK257685, MK257687, MK257688, MK257689, MK257696, MK257697, and MK257698 was 99.81%. These sequences had a mismatch in nucleotide 433 (G/T), which caused the substitution of G342A for G342V and clustered them in 2 different branches (Supplementary Fig. 1). In addition, a synonymous mutation in nucleotide 447 (A/G) clustered MK257686 in different branches, despite its 100% similarity with sensitive samples (MK257694 and MK257699) at amino acid level (Fig. 3).

## Discussion

Since 1967, OP and CB insecticides have widely been used in vector control, particularly in the malaria vector control program in Iran (Abbasi et al. 2019). During 1984–1994, 35,810,000 kg of pesticides, including insecticides, fungicides, and herbicides, have been exploited in agriculture in the country, but it has recently reduced to 25,000,000 kg for 1 single year. The most crucial reason for the spread of insecticide resistance is the overuse or intensive use of these compounds (Başkurt et al. 2011, Arich et al. 2021, Wang et al. 2022). There is very minimal information on the housefly resistance to insecticides in Iran. Almost all surveys conducted in Iran on *M. domestica* have devoted their attention to the contamination of housefly with various pathogens such as fungi and bacteria (Davari et al. 2012, Hemmatinezhad et al. 2015, Nazari et al. 2017); however, knockdown resistance (*kdr*) in housefly has lately been studied (Kamdar et al. 2019, Riaz et al. 2022, Roca-Acevedo et al. 2023). The current study has examined polymorphisms in the ACE gene of houseflies collected from Iran. L260-G342-Y407 and L260-G342-F407 combinations were novel and specific for the Iranian housefly population. However, L260-G342-F407 was reported from Korean *M. domestica* population in 2004 (Im et al. 2004). Together with G445A, these mutations have been described as OP and CB resistance-associated mutations (Kozaki et al. 2001a, Walsh et al. 2001).

The variety of well-known and potential insecticide-resistant ACE alleles varies from one region to another, though there may overlap. The partial amplification and sequencing of the ACE gene in houseflies collected from 16 provinces in Turkey (the neighboring country to Iran) showed that L/V260-A/G342-F/Y47 was the highest combination (Başkurt et al. 2011). In comparison, the prevalent combination in Iranian samples was V260-V342-Y407 (Allele II), with 40% prevalence rate. Alleles I, III, and V in the Iranian population were compatible with alleles from Turkey (Başkurt et al. 2011) and United States (Kozaki et al. 2009) (Table 2). The V11, a US population allele, was not detected in Iranian and Turkish populations. Instead, alleles II and IV, specific to the Iranian population, were not found in the United States, Turkey, Japan, Denmark, and United Kingdom (Walsh et al. 2001, Kristensen et al. 2006, Kozaki et al. 2009, Başkurt et al. 2011). Alleles II and IV were reported as novel and specific alleles to the Iranian *M. domestica* population. Interestingly, alleles II and III in Turkish population (Başkurt et al. 2011) were the same as L260-V342-Y407. Therefore, it could be speculated that the underlying nucleotide sequences for alleles II and III are different, even though both result in L260-A342-Y407.

Overall, the current study identified 5 alleles. Phylogenetic analysis revealed multiple origins for I, II, and III alleles and a single origin for IV and V alleles (Fig. 4). According to Kozaki et al. (2009), F407Y and G342A have multiple origins, while A316S, G342V, and V260L have a single origin (Kozaki et al. 2009). The variation observed in the ACE gene in the Iranian housefly population could be justified by the variety and pressure of insecticides used for vector control and crop protection in the country. Therefore, it could be deduced that I, II, and III alleles have evolved independently in various geographic locations due to similar selection pressure.

Insecticide resistance levels are varying in the presence of different mutations (Kozaki et al. 2001a, b, Walsh et al. 2001). Sensitivity to dichlorvos and bendiocarb decreased 58-fold and 85-fold in the presence of G326V mutation, respectively (Walsh et al. 2001). However, 342V mutation has a key role in insensitivity to dichlorvos than other mutations (Walsh et al. 2001). On the other hand, combination of AChE mutations often causes higher insecticide resistance than single mutation (Walsh et al. 2001, Liming et al. 2006, Wang et al. 2012). In the current study, 4 reported mutations were categorized into 5 different combinations containing 1–3 mutations (Table 2). It has been reported that G342V+F407Y combination increased dichlorvos insensitivity 240-fold (Walsh et al. 2001). Based on the findings of the current study, it could be postulated that the level of resistance to dichlorvos is high in study area and need further studies on the relative insensitivity and ACE mutation combinations roles in insecticide resistance management.

## Conclusions

Iranian housefly population is comprised of 5 insensitive and sensitive alleles. Such allelic variations have also been reported in Turkey (Başkurt et al. 2011) and laboratory and field populations in the United States (Kozaki et al. 2009). Each allele is responsible for specific resistance to certain insecticides (Menozzi et al. 2004); therefore, the emergence of multiple alleles in the field population might arise from continuous and prolonged use of the same insecticides in the Iranian community. However, Py and OP combination is still successful against housefly. The development of an effective control strategy for such organizations seems to be difficult. Combination use of different groups of insecticides could serve as a strategy for combating resistance and inhibiting its appearance.

Altogether, the genetic basis of insecticide resistance is complex and multifactorial, involving interactions between the environment, housefly, and insecticides themselves. The current study was conducted in a limited area with a small number of samples; however, this survey could be implemented in a whole country and even the neighbor countries with a larger sample. Continuous research is required to better understand between the environment, housefly, insecticides, and develop effective strategies for the management of housefly resistance to insecticides.

## Supplementary Material

iead054_suppl_Supplementary_Figure_S1

## Data Availability

The datasets generated and/or analyzed during the current study are available in the GenBank, European Molecular Biology Laboratory (EMBL), and DNA Data Bank of Japan (DDBJ) repository, GenBank IDs: MK257685-MK257704 (https://www.ncbi.nlm.nih.gov/nuccore).
