# Supplementary material for: Molecular analysis of acetylcholinesterase gene in field-collected populations of Musca domestica (Diptera: Muscidae) in Northwestern Iran
Source: J Insect Sci. 2023 Jul 22;23(4):9. doi: 10.1093/jisesa/iead054 (PMC10362979; doi:10.1093/jisesa/iead054)
Supplement: iead054_suppl_Supplementary_Figure_S1 [file iead054_suppl_supplementary_figure_s1.docx]

**Supp. Fig.1:**

#AF281161aabys CATCTAAAAC CGATCAGGAC CATTTAATAC ATAGCGCAAC GCCACAGAAC ACCACAAATG [ 60]

MK257685 .......... .......... .......... .......... .......... .......... [ 60]

MK257686 .......... .......... .......... .......... .......... .......... [ 60]

MK257687 .......... .......... .......... .......... .......... .......... [ 60]

MK257688 .......... .......... .......... .......... .......... .......... [ 60]

MK257689 .......... .......... .......... .......... .......... .......... [ 60]

MK257690 .......... .......... .......... .......... .......... .......... [ 60]

MK257691 .......... .......... .......... .......... .......... .......... [ 60]

MK257692 .......... .......... .......... .......... .......... .......... [ 60]

MK257693 .......... .......... .......... .......... .......... .......... [ 60]

MK257694 .......... .......... .......... .......... .......... .......... [ 60]

MK257695 .......... .......... .......... .......... .......... .......... [ 60]

MK257696 .......... .......... .......... .......... .......... .......... [ 60]

MK257697 .......... .......... .......... .......... .......... .......... [ 60]

MK257698 .......... .......... .......... .......... .......... .......... [ 60]

MK257699 .......... .......... .......... .......... .......... .......... [ 60]

MK257700 .......... .......... .......... .......... .......... .......... [ 60]

MK257701 .......... .......... .......... .......... .......... .......... [ 60]

MK257702 .......... .......... .......... .......... .......... .......... [ 60]

MK257703 .......... .......... .......... .......... .......... .......... [ 60]

MK257704 .......... .......... .......... .......... .......... .......... [ 60]

#Md-v1FJ174253 .--------- ---------- ---------- ---------- ---------- ---------- [ 60]

#Md-v2FJ174254 .--------- ---------- ---------- ---------- ---------- ---------- [ 60]

#Md-v3FJ174255 .--------- ---------- ---------- ---------- ---------- ---------- [ 60]

#Md-v4FJ174256 .--------- ---------- ---------- ---------- ---------- ---------- [ 60]

#Md-v5FJ174257 .--------- ---------- ---------- ---------- ---------- ---------- [ 60]

#Md-v6FJ174258 .--------- ---------- ---------- ---------- ---------- ---------- [ 60]

#Md-v7FJ174259 .--------- ---------- ---------- ---------- ---------- ---------- [ 60]

#Md-v8FJ174260 .--------- ---------- ---------- ---------- ---------- ---------- [ 60]

#Md-v9FJ174261 .--------- ---------- ---------- ---------- ---------- ---------- [ 60]

#Md-v10FJ174262 .--------- ---------- ---------- ---------- ---------- ---------- [ 60]

#Md-v11FJ174263 .--------- ---------- ---------- ---------- ---------- ---------- [ 60]

#Md-v12FJ174264 .--------- ---------- ---------- ---------- ---------- ---------- [ 60]

#Md-v13FJ174265 .--------- ---------- ---------- ---------- ---------- ---------- [ 60]

#Md-v14FJ174266 .--------- ---------- ---------- ---------- ---------- ---------- [ 60]

#Md-v15FJ174267 .--------- ---------- ---------- ---------- ---------- ---------- [ 60]

#AF281161aabys GTTTGCCTAT ATTAATATGG ATTTATGGCG GTGGCTTTAT GACTGGCTCA GCCACATTGG [120]

MK257685 .......... .......... .......... .......... .......... .......... [120]

MK257686 .......... .......... .......... .......... .......... .......... [120]

MK257687 .......... .......... .......... .......... .......... .......... [120]

MK257688 .......... .......... .......... .......... .......... .......... [120]

MK257689 .......... .......... .......... .......... .......... .......... [120]

MK257690 .......... .......... .......... .......... .......... .......... [120]

MK257691 .......... .......... .......... .......... .......... .......... [120]

MK257692 .......... .......... .......... .......... .......... .......... [120]

MK257693 .......... .......... .......... .......... .......... .......... [120]

MK257694 .......... .......... .......... .......... .......... .......... [120]

MK257695 .......... .......... .......... .......... .......... .......... [120]

MK257696 .......... .......... .......... .......... .......... .......... [120]

MK257697 .......... .......... .......... .......... .......... .......... [120]

MK257698 .......... .......... .......... .......... .......... .......... [120]

MK257699 .......... .......... .......... .......... .......... .......... [120]

MK257700 .......... .......... .......... .......... .......... .......... [120]

MK257701 .......... .......... .......... .......... .......... .......... [120]

MK257702 .......... .......... .......... .......... .......... .......... [120]

MK257703 .......... .......... .......... .......... .......... .......... [120]

MK257704 .......... .......... .......... .......... .......... .......... [120]

#Md-v1FJ174253 ---------- ---------- ---------- ---------- ---------- ---------- [120]

#Md-v2FJ174254 ---------- ---------- ---------- ---------- ---------- ---------- [120]

#Md-v3FJ174255 ---------- ---------- ---------- ---------- ---------- ---------- [120]

#Md-v4FJ174256 ---------- ---------- ---------- ---------- ---------- ---------- [120]

#Md-v5FJ174257 ---------- ---------- ---------- ---------- ---------- ---------- [120]

#Md-v6FJ174258 ---------- ---------- ---------- ---------- ---------- ---------- [120]

#Md-v7FJ174259 ---------- ---------- ---------- ---------- ---------- ---------- [120]

#Md-v8FJ174260 ---------- ---------- ---------- ---------- ---------- ---------- [120]

#Md-v9FJ174261 ---------- ---------- ---------- ---------- ---------- ---------- [120]

#Md-v10FJ174262 ---------- ---------- ---------- ---------- ---------- ---------- [120]

#Md-v11FJ174263 ---------- ---------- ---------- ---------- ---------- ---------- [120]

#Md-v12FJ174264 ---------- ---------- ---------- ---------- ---------- ---------- [120]

#Md-v13FJ174265 ---------- ---------- ---------- ---------- ---------- ---------- [120]

#Md-v14FJ174266 ---------- ---------- ---------- ---------- ---------- ---------- [120]

#Md-v15FJ174267 ---------- ---------- ---------- ---------- ---------- ---------- [120]

#AF281161aabys ACATTTACAA CGCTGAGATT ATGTCGGCCG TGGGCAATGT GATCGTTGCC TCGTTCCAGT [180]

MK257685 .......... ...A...... .......... .......... .......... .......... [180]

MK257686 .......... ...G...... .......... .......... .......... .......... [180]

MK257687 .......... ...A...... .......... .......... .......... .......... [180]

MK257688 .......... ...A...... .......... .......... .......... .......... [180]

MK257689 .......... ...A...... .......... .......... .......... .......... [180]

MK257690 .......... ...A...... .......... .......... .......... .......... [180]

MK257691 .......... ...A...... .......... .......... .......... .......... [180]

MK257692 .......... ...A...... .......... .......... .......... .......... [180]

MK257693 .......... ...A...... .......... .......... .......... .......... [180]

MK257694 .......... ...A...... .......... .......... .......... .......... [180]

MK257695 .......... ...A...... .......... .......... .......... .......... [180]

MK257696 .......... ...A...... .......... .......... .......... .......... [180]

MK257697 .......... ...A...... .......... .......... .......... .......... [180]

MK257698 .......... ...A...... .......... .......... .......... .......... [180]

MK257699 .......... ...A...... .......... .......... .......... .......... [180]

MK257700 .......... ...A...... .......... .......... .......... .......... [180]

MK257701 .......... ...A...... .......... .......... .......... .......... [180]

MK257702 .......... ...A...... .......... .......... .......... .......... [180]

MK257703 .......... ...A...... .......... .......... .......... .......... [180]

MK257704 .......... ...A...... .......... .......... .......... .......... [180]

#Md-v1FJ174253 ---------- ---------- ---------. .......... .......... .......... [180]

#Md-v2FJ174254 ---------- ---------- ---------. .......... .......... .......... [180]

#Md-v3FJ174255 ---------- ---------- ---------. .......... .......... .......... [180]

#Md-v4FJ174256 ---------- ---------- ---------. .......... .......... .......... [180]

#Md-v5FJ174257 ---------- ---------- ---------. .......... .......... .......... [180]

#Md-v6FJ174258 ---------- ---------- ---------. .......... .......... .......... [180]

#Md-v7FJ174259 ---------- ---------- ---------. .......... .......... .......... [180]

#Md-v8FJ174260 ---------- ---------- ---------. .......... .......... .......... [180]

#Md-v9FJ174261 ---------- ---------- ---------. .......... .......... .......... [180]

#Md-v10FJ174262 ---------- ---------- ---------. .......... .......... .......... [180]

#Md-v11FJ174263 ---------- ---------- ---------. .......... .......... .......... [180]

#Md-v12FJ174264 ---------- ---------- ---------. .......... .......... .......... [180]

#Md-v13FJ174265 ---------- ---------- ---------. .......... .......... .......... [180]

#Md-v14FJ174266 ---------- ---------- ---------. .......... .......... .......... [180]

#Md-v15FJ174267 ---------- ---------- ---------. .......... .......... .......... [180]

1226

#AF281161aabys ATCGAGTCGG TGCATTTGGG TTTCTACATC TTTCACCGGT TATGCCAGGT TTTGAAGAAG [240]

MK257685 .......... .......... .......... .......... .......... .......... [240]

MK257686 .......... .......... .......... .......... .......... .......... [240]

MK257687 .......... .......... .......... .......... .......... .......... [240]

MK257688 .......... .......... .......... .......... .......... .......... [240]

MK257689 .......... .......... .......... .......... .......... .......... [240]

MK257690 .....C.... .......... .......... .......... .......... .......... [240]

MK257691 .......... .......... .......... .......... .......... .......... [240]

MK257692 .....C.... .......... .......... .......... .......... .......... [240]

MK257693 .....C.... .......... .......... .......... .......... .......... [240]

MK257694 .......... .......... .......... .......... .......... .......... [240]

MK257695 .....C.... .......... .......... .......... .......... .......... [240]

MK257696 .......... .......... .......... .......... .......... .......... [240]

MK257697 .......... .......... .......... .......... .......... .......... [240]

MK257698 .......... .......... .......... .......... .......... .......... [240]

MK257699 .......... .......... .......... .......... .......... .......... [240]

MK257700 .....C.... .......... .......... .......... .......... .......... [240]

MK257701 .......... .......... .......... .......... .......... .......... [240]

MK257702 .......... .......... .......... .......... .......... .......... [240]

MK257703 .......... .......... .......... .......... .......... .......... [240]

MK257704 .....C.... .......... .......... .......... .......... .......... [240]

#Md-v1FJ174253 .......... .......... .......... .......... .......... .......... [240]

#Md-v2FJ174254 .......... .......... .......... .......... .......... .......... [240]

#Md-v3FJ174255 .......... .......... .......... .......... .......... .......... [240]

#Md-v4FJ174256 .......... .......... .......... .......... .......... .......... [240]

#Md-v5FJ174257 .......... .......... .......... .......... .......... .......... [240]

#Md-v6FJ174258 .......... .......... .......... .......... .......... .......... [240]

#Md-v7FJ174259 .......... .......... .......... .......... .......... .......... [240]

#Md-v8FJ174260 .......... .......... .......... .......... .......... .......... [240]

#Md-v9FJ174261 .......... .......... .......... .......... .......... .......... [240]

#Md-v10FJ174262 .......... .......... .......... .......... .......... .......... [240]

#Md-v11FJ174263 .......... .......... .......... .......... .......... .......... [240]

#Md-v12FJ174264 .......... .......... .......... .......... .......... .......... [240]

#Md-v13FJ174265 .......... .......... .......... .......... .......... .......... [240]

#Md-v14FJ174266 .......... .......... .......... .......... .......... .......... [240]

#Md-v15FJ174267 .....C.... .......... .......... .......... .......... .......... [240]

#AF281161aabys AAGCTCCCGG CAACGTGGGC CTTTGGGATC AGGCCTTGGC TTTGCGTTGG CTGAAGGAGA [300]

MK257685 .......... .........T .......... .......... C......... .......... [300]

MK257686 .......... .......... .......... .......... C......... .......... [300]

MK257687 .......... .........T .......... .......... C......... .......... [300]

MK257688 .......... .........T .......... .......... C......... .......... [300]

MK257689 .......... .........T .......... .......... C......... .......... [300]

MK257690 .......... .......... .......... .......... C......... ..T....... [300]

MK257691 .......... .......... .......... .......... C......... ..T....... [300]

MK257692 .......... .......... .......... .......... C......... ..T....... [300]

MK257693 .......... .......... .......... .......... C......... ..T....... [300]

MK257694 .......... .......... .......... .......... C......... .......... [300]

MK257695 .......... .......... .......... .......... C......... ..T....... [300]

MK257696 .......... .........T .......... .......... C......... .......... [300]

MK257697 .......... .........T .......... .......... C......... .......... [300]

MK257698 .......... .........T .......... .......... C......... .......... [300]

MK257699 .......... .......... .......... .......... C......... .......... [300]

MK257700 .......... .........T .......... .......... C......... .......... [300]

MK257701 .......... .........T .......... .......... C......... .......... [300]

MK257702 .......... .........T .......... .......... C......... .......... [300]

MK257703 .......... .........T .......... .......... C......... .......... [300]

MK257704 .......... .......... .......... .......... C......... ..T....... [300]

#Md-v1FJ174253 .......... .......... .......... .......... .......... .......... [300]

#Md-v2FJ174254 .......... .........T .......... .......... C......... .......... [300]

#Md-v3FJ174255 .......... .........T .......... .......... C......... .......... [300]

#Md-v4FJ174256 .......... .......... .......... .......... C......... .......... [300]

#Md-v5FJ174257 .......... .......... .......... .......... C......... .......... [300]

#Md-v6FJ174258 .......... ......T... .......... .......... C......... ........A. [300]

#Md-v7FJ174259 .......... .......... .......... .......... C......... .......... [300]

#Md-v8FJ174260 .......... .......... .......... .......... C......... .......... [300]

#Md-v9FJ174261 .......... .......... .......... .......... C......... .......... [300]

#Md-v10FJ174262 .......... .........T .......... .......... C......... .......... [300]

#Md-v11FJ174263 .......... .........T .......... .......... C......... .......... [300]

#Md-v12FJ174264 .......... .........T .......... .......... C......... .......... [300]

#Md-v13FJ174265 .......... .......... .......... ....A..... C......... .......... [300]

#Md-v14FJ174266 .......... .......... .......... .......... C......... ..T....... [300]

#Md-v15FJ174267 .......... .......... .......... .......... C......... ..T....... [300]

#AF281161aabys ATGCCCGTGC ATTTGGCGGC AATCCGGAAT GGATGACGCT GTTTGGTGAA TCGGCTGGTT [360]

MK257685 .......... .......... .......... .......... .......... .......... [360]

MK257686 .......... .......... .......... .......... .......... .......... [360]

MK257687 .......... .......... .......... .......... .......... .......... [360]

MK257688 .......... .......... .......... .......... .......... .......... [360]

MK257689 .......... .......... .......... .......... .......... .......... [360]

MK257690 .......... .......... .......... .......... .......... .......... [360]

MK257691 .......... .......... .......... .......... .......... .......... [360]

MK257692 .......... .......... .......... .......... .......... .......... [360]

MK257693 .......... .......... .......... .......... .......... .......... [360]

MK257694 .......... .......... .......... .......... .......... .......... [360]

MK257695 .......... .......... .......... .......... .......... .......... [360]

MK257696 .......... .......... .......... .......... .......... .......... [360]

MK257697 .......... .......... .......... .......... .......... .......... [360]

MK257698 .......... .......... .......... .......... .......... .......... [360]

MK257699 .......... .......... .......... .......... .......... .......... [360]

MK257700 .......... .......... .......... .......... .......... .......... [360]

MK257701 .......... .......... .......... .......... .......... .......... [360]

MK257702 .......... .......... .......... .......... .......... .......... [360]

MK257703 .......... .......... .......... .......... .......... .......... [360]

MK257704 .......... .......... .......... .......... .......... .......... [360]

#Md-v1FJ174253 .......... .......... .......... .......... .......... .......... [360]

#Md-v2FJ174254 .......... .......... .......... .......... .......... .......... [360]

#Md-v3FJ174255 .......... .......... .......... .......... .......... .......... [360]

#Md-v4FJ174256 .......... .......... .......... .......... .......... .......... [360]

#Md-v5FJ174257 .......... .......... .......... .......... .......... .......... [360]

#Md-v6FJ174258 .......... .......... .......... .......T.. .......... .......... [360]

#Md-v7FJ174259 .......... .......... .......... .......... .......... .......... [360]

#Md-v8FJ174260 .......... .......... .......... .......... .......... .......... [360]

#Md-v9FJ174261 .......... .......... .......... .......... .......... .......... [360]

#Md-v10FJ174262 .......... .......... .......... .......... .......... .......... [360]

#Md-v11FJ174263 .......... .......... .......... .......... .......... ...T...... [360]

#Md-v12FJ174264 .......... .......... .......... .......... .......... .......... [360]

#Md-v13FJ174265 .......... .......... .......... .......... .......... .......... [360]

#Md-v14FJ174266 .......... .......... .......... .......... .......... .......... [360]

#Md-v15FJ174267 .......... .......... .......... .......... .......... .......... [360]

#AF281161aabys CGAGTTCCGT GAATGCTCAA CTGATGTCGC CGGTAACGCG TGGCCTGGTC AAACGTGGCA [420]

MK257685 .......... .......... .......... .......... .......... .......... [420]

MK257686 .......... .......... .......... .......... .......... .......... [420]

MK257687 .......... .......... .......... .......... .......... .......... [420]

MK257688 .......... .......... .......... .......... .......... .......... [420]

MK257689 .......... .......... .......... .......... .......... .......... [420]

MK257690 .......... .......... .......... .......... .......... .......... [420]

MK257691 .......... .......... .......... .......... .......... .......... [420]

MK257692 .......... .......... .......... .......... .......... .......... [420]

MK257693 .......... .......... .......... .......... .......... .......... [420]

MK257694 .......... .......... .......... .......... .......... .......... [420]

MK257695 .......... .......... .......... .......... .......... .......... [420]

MK257696 .......... .......... .......... .......... .......... .......... [420]

MK257697 .......... .......... .......... .......... .......... .......... [420]

MK257698 .......... .......... .......... .......... .......... .......... [420]

MK257699 .......... .......... .......... .......... .......... .......... [420]

MK257700 .......... .......... .......... .......... .......... .......... [420]

MK257701 .......... .......... .......... .......... .......... .......... [420]

MK257702 .......... .......... .......... .......... .......... .......... [420]

MK257703 .......... .......... .......... .......... .......... .......... [420]

MK257704 .......... .......... .......... .......... .......... .......... [420]

#Md-v1FJ174253 .......... .......... .......... .......... .......... .......... [420]

#Md-v2FJ174254 .......... .......... .......... .......... .......... .......... [420]

#Md-v3FJ174255 .......... .......... .......... .......... .......... .......... [420]

#Md-v4FJ174256 .......... .......... .......... .......... .......... .......... [420]

#Md-v5FJ174257 .......... .......... .......... .......... .......... .......... [420]

#Md-v6FJ174258 .......... .......... .......... .......... .......... .......... [420]

#Md-v7FJ174259 .......... .......... .......... .......... .......... .......... [420]

#Md-v8FJ174260 .......... .......... .......... .......... .......... .......... [420]

#Md-v9FJ174261 .......... .......... .......... .......... .......... .......... [420]

#Md-v10FJ174262 .......... .......... .......... .......... .......... .......... [420]

#Md-v11FJ174263 .......... .......... .......... .......... .......... .......... [420]

#Md-v12FJ174264 .......... .......... .......... .......... .......... .......... [420]

#Md-v13FJ174265 .......... .......... .......... .......... .......... .......... [420]

#Md-v14FJ174266 .......... .......... .......... .......... .......... .......... [420]

#Md-v15FJ174267 .......... .......... .......... .......... .......... .......... [420]

1473

#AF281161aabys TGATGCAGTC GGGCACAATG AATGCTCCCT GGAGCCACAT GACTTCAGAG AAGGCGGTTG [480]

MK257685 .......... ..T....... .......... .......... ...A...... .......... [480]

MK257686 .......... .......... .......... .......... ...A...... .......... [480]

MK257687 .......... ..T....... .......... .......... ...A...... .......... [480]

MK257688 .......... ..T....... .......... .......... ...A...... .......... [480]

MK257689 .......... ..T....... .......... .......... ...A...... .......... [480]

MK257690 .......... ..C....... .......... .......... ...A...... .......... [480]

MK257691 .......... ..T....... .......... .......... ...A...... .......... [480]

MK257692 .......... ..C....... .......... .......... ...A...... .......... [480]

MK257693 .......... ..C....... .......... .......... ...A...... .......... [480]

MK257694 .......... .......... .......... .......... ...A...... .......... [480]

MK257695 .......... .......... .......... .......... ...A...... .......... [480]

MK257696 .......... ..T....... .......... .......... ...A...... .......... [480]

MK257697 .......... ..T....... .......... .......... ...A...... .......... [480]

MK257698 .......... ..T....... .......... .......... ...A...... .......... [480]

MK257699 .......... .......... .......... .......... ...A...... .......... [480]

MK257700 .......... .......... .......... .......... ...A...... .......... [480]

MK257701 .......... ..C....... .......... .......... ...A...... .......... [480]

MK257702 .......... ..C....... .......... .......... ...A...... .......... [480]

MK257703 .......... ..C....... .......... .......... ...A...... .......... [480]

MK257704 .......... .......... .......... .......... ...A...... .......... [480]

#Md-v1FJ174253 .......... .......... .......... .......... .......... .......... [480]

#Md-v2FJ174254 .......... .......... .......... .......... ...A...... .......... [480]

#Md-v3FJ174255 .......... .......... .......... .......... ...A...... .......... [480]

#Md-v4FJ174256 .......... .......... .......... .......... .......... .......... [480]

#Md-v5FJ174257 .......... .......... .......... .......... .......... .......... [480]

#Md-v6FJ174258 .......... .......... .......... .......... ...A...... .......... [480]

#Md-v7FJ174259 .......... .......... .......... .......... ...A...... .......... [480]

#Md-v8FJ174260 .......... .......... .......... .......... ...A...... .......... [480]

#Md-v9FJ174261 .......... .......... .......... .......... ...A...... .......... [480]

#Md-v10FJ174262 .......... ..C....... .......... .......... ...A...... .......... [480]

#Md-v11FJ174263 .......... ..C....... .......... .......... ...A...... .......... [480]

#Md-v12FJ174264 .......... .......... .......... .......... ...A...... .......... [480]

#Md-v13FJ174265 .......... ...T...... .......... .......... ...A...... .......... [480]

#Md-v14FJ174266 .......... ..T....... .......... .......... ...A...... .......... [480]

#Md-v15FJ174267 .......... ..C....... .......... .......... ...A...... .......... [480]

#AF281161aabys AAATTGGTAA AGCTTTGGTA AATGACTGTA ACTGTAATGC CTCATTGTTA CCGG------ [540]

MK257685 .......... .......... .......... .......... .......... ....TAAGCA [540]

MK257686 .......... .......... .......... .......... .......... ....TAAGCA [540]

MK257687 .......... .......... .......... .......... .......... ....TAAGCA [540]

MK257688 .......... .......... .......... .......... .......... ....TAAGCA [540]

MK257689 .......... .......... .......... .......... .......... ....TAAGCA [540]

MK257690 .......... .......... .......... .......... .......... ....TAAGCA [540]

MK257691 .......... .......... .......... .......... .......... ....TAAGCA [540]

MK257692 .......... .......... .......... .......... .......... ....TAAGCA [540]

MK257693 .......... .......... .......... .......... .......... ....TAAGCA [540]

MK257694 .......... .......... .......... .......... .......... ....TAAGCA [540]

MK257695 .......... .......... .......... .......... .......... ....TAAGCA [540]

MK257696 .......... .......... .......... .......... .......... ....TAAGCA [540]

MK257697 .......... .......... .......... .......... .......... ....TAAGCA [540]

MK257698 .......... .......... .......... .......... .......... ....TAAGCA [540]

MK257699 .......... .......... .......... .......... .......... ....TAAGCA [540]

MK257700 .......... .......... .......... .......... .......... ....TAAGCA [540]

MK257701 .......... .......... .......... .......... .......... ....TAAGCA [540]

MK257702 .......... .......... .......... .......... .......... ....TAAGCA [540]

MK257703 .......... .......... .......... .......... .......... ....TAAGCA [540]

MK257704 .......... .......... .......... .......... .......... ....TAAGCA [540]

#Md-v1FJ174253 .......... .......... .......... .......... .......... ....TAAGCA [540]

#Md-v2FJ174254 .......... .......... .......... .......... .......... ....TAAGCA [540]

#Md-v3FJ174255 .......... .......... .......... .......... .......... ....TAAGCA [540]

#Md-v4FJ174256 .......... .......... .......... .......... .......... ....TAAGCA [540]

#Md-v5FJ174257 .......... .......... .......... .......... .......... ....TAAGCA [540]

#Md-v6FJ174258 .......... .......... .......... .......... .......... ....TAAGCA [540]

#Md-v7FJ174259 .......... .......... .......... .......... .......... ....TAAGCA [540]

#Md-v8FJ174260 .......... .......... .......... .......... .......... ....TAAGCA [540]

#Md-v9FJ174261 .......... .......... .......... .......... .......... ....TAAGCA [540]

#Md-v10FJ174262 .......... .......... .......... .......... .......... ....TAAGCA [540]

#Md-v11FJ174263 .......... .......... .......... .......... .......... ....TAAGCA [540]

#Md-v12FJ174264 .......... .......... .......... .......... .......... ....TAAGCA [540]

#Md-v13FJ174265 .......... .......... .......... .......... .......... ....TAAGCA [540]

#Md-v14FJ174266 .......... .......... .......... .......... .......... ....TAAGCA [540]

#Md-v15FJ174267 .......... .......... .......... .......... .......... ....TAAGCA [540]

#AF281161aabys ---------- ---------- ---------- ---------- ---------- ---------- [600]

MK257685 GTGGAGTATT TATGCGACTA TACGAATTTT TCTTTGCTGA TGAATTT--- TATTTTCCTC [600]

MK257686 GTGGAGTATT TATGCGATTA TACGAATTTT TCTTTGCTGA TGCATTTATT TATTTTCCTG [600]

MK257687 GTGGAGTATT TATGCGACTA TACGAATTTT TCTTTGCTGA TGAATTT--- TATTTTCCTC [600]

MK257688 GTGGAGTATT TATGCGACTA TACGAATTTT TCTTTGCTGA TGAATTT--- TATTTTCCTC [600]

MK257689 GTGGAGTATT TATGCGACTA TACGAATTTT TCTTTGCTGA TGAATTT--- TATTTTCCTC [600]

MK257690 GTGGAGTATT TATGCGATTA CGAGAATTTT TCTTTGCTGA TGAATTTT-- -ATTTCCCTC [600]

MK257691 GTGGAGTATT TATGCGATTA TACGAATTTT TCTTTGCTGA TGAATTT--- TATTTTCCTC [600]

MK257692 GTGGAGTATT TATGCGATTA CGAGAATTTT TCTTTGCTGA TGAATTTT-- -ATTTCCCTC [600]

MK257693 GTGGAGTATT TATGCGATTA CGAGAATTTT TCTTTGCTGA TGAATTTT-- -ATTTCCCTC [600]

MK257694 GTGGAGTATT TATGCGATTA TACGAATTTT TCTTTGCTGA TGAATTT--- TATTTTCCTC [600]

MK257695 GTGGAGTATT TATGCGATTA CGAGAATTTT TCTTTGCTGA TGAATTTT-- -ATTTCCCTC [600]

MK257696 GTGGAGTATT TATGCGACTA TACGAATTTT TCTTTGCTGA TGAATTT--- TATTTTCCTC [600]

MK257697 GTGGAGTATT TATGCGACTA TACGAATTTT TCTTTGCTGA TGAATTT--- TATTTTCCTC [600]

MK257698 GTGGAGTATT TATGCGATTA TACGAATTTT TCTTTGCTGA TGAATTT--- TATTTTCCTC [600]

MK257699 GTGGAGTATT TATGCGATTA TAAGAATTTT TCTTTGATGA TGAATTT--- TATTTTCCTC [600]

MK257700 GTGGAGTATT TATGCGATTA TACGAATTTT TCTTTGCTGA TGAATTT--- TATTTTCCTC [600]

MK257701 GTGGAGTATT TATGCGACTA TACGAATTTT TCTTTGCTGA TGAATTT--- TATTTTCCTC [600]

MK257702 GTGGAGTATT TATGCGACTA TACGAATTTT TCTTTGCTGA TGAATTT--- TATTTTCCTC [600]

MK257703 GTGGAGTATT TATGCGACTA TACGAATTTT TCTTTGCTGA TGAATTT--- TATTTTCCTC [600]

MK257704 GTGGAGTATT TATGCGATTA CGAGAATTTT TCTTTGCTGA TGAATTTT-- -ATTTCCCTC [600]

#Md-v1FJ174253 GTGGAGTATT TATGCGATTA TACGAATTTT TCTTTGCTGA TGAATTTT-- -ATTTTCCTC [600]

#Md-v2FJ174254 GTGGAGTATT TATGCGACTA TACGAATTTT TCTTTGCTGA TGAATTT--- TATTTTCCTC [600]

#Md-v3FJ174255 GTGGAGTATT TATGCGATTA TACGAATTTT TCTTTGCTGA TGCATTTATT TATTTTCCTG [600]

#Md-v4FJ174256 GTGGAGTATT TATGCGATTA TACGAATTTT TCTTTGCTGA TGAATTTTTT TATTTTCCTC [600]

#Md-v5FJ174257 GTGGAGTATT TATGCGATTA TACGAATTTT TCTTTGCTGA TGAATTTTAT TATTTTCCTG [600]

#Md-v6FJ174258 GTGGAGTATT TATGCGATTA TACGAATTTT TCTTTGCTGA TGAATTT--- TATTTCCCTC [600]

#Md-v7FJ174259 GTGGAGTATT TATGCGATTA TACGAATTTT TCTTTGCTGA TGCATTTATT TATTTTCCTG [600]

#Md-v8FJ174260 GTGGAGTATT TATGCGATTA TACGAATTTT TCTTTGCTGA TGAATTT--- TATTTTCCTC [600]

#Md-v9FJ174261 GTGGAGTATT TATGCGATTA CGAGAATTTT TCTTTGCTGA TGAATTTT-- -ATTTCCCTC [600]

#Md-v10FJ174262 GTGGAGTATT TATGCGACTA TACGAATTTT TCTTTGCTGA TGAATTT--- TATTTTCCTC [600]

#Md-v11FJ174263 GTGGAGTATT TATGCGACTA TACGAATTTT TCTTTGCTGA TGAATTT--- TATTTTCCTC [600]

#Md-v12FJ174264 GTGGAGTATT TATGCGATTA TACGAATTTT TCTTTGCTGA TGAATTT--- TATTTTCCTG [600]

#Md-v13FJ174265 GTGGAGTATT TATGCGATTA TACGAATTTT TCTTTGCTGA TGAATTT--- TATTTTCCTC [600]

#Md-v14FJ174266 GTGGAGTATT TATGCGATTA CGAGAATTTT TCTTTGCTGA TGAATTTT-- -ATTTCCCTC [600]

#Md-v15FJ174267 GTGGAGTATT TATGCGATTA CGAGAATTTT TCTTTGCTGA TGAATTTT-- -ATTTCCCTC [600]

#AF281161aabys ---------- ---------- --AAAATCCA CAAGCTGTCA TGGCTTGCAT GCGACAGGTT [660]

MK257685 TTTTTTGTGT TTTCAACTTA GG........ .......... .......... .......... [660]

MK257686 TTCTTTGTGT TTTCAACTTA GG........ .......... .......... .......... [660]

MK257687 TTTTTTGTGT TTTCAACTTA GG........ .......... .......... .......... [660]

MK257688 TTTTTTGTGT TTTCAACTTA GG........ .......... .......... .......... [660]

MK257689 TTTTTTGTGT TTTCAACTTA GG........ .......... .......... .......... [660]

MK257690 TTCTTTGTGT TCTCAACTTA GG........ .......... .......... .......... [660]

MK257691 TTTTTTGTGT TTTCAACTTA GG........ .......... .......... .......... [660]

MK257692 TTCTTTGTGT TCTCAACTTA GG........ .......... .......... .......... [660]

MK257693 TTCTTTGTGT TCTCAACTTA GG........ .......... .......... .......... [660]

MK257694 TTCTTTGTGT TTTCAACTTA GG........ .......... .......... .......... [660]

MK257695 TTCTTTGTGT TCTCAACTTA GG........ .......... .......... .......... [660]

MK257696 TTTTTTGTGT TTTCAACTTA GG........ .......... .......... .......... [660]

MK257697 TTTTTTGTGT TTTCAACTTA GG........ .......... .......... .......... [660]

MK257698 TTCTTTGTGT TTTCAACTTA GG........ .......... .......... .......... [660]

MK257699 TTCTTTGTGT TTTCAACTTA GG........ .......... .......... .......... [660]

MK257700 TTCTTTGTGT TTTCAACTTA GG........ .......... .......... .......... [660]

MK257701 TTTTTTGTGT TTTCAACTTA GG........ .......... .......... .......... [660]

MK257702 TTTTTTGTGT TTTCAACTTA GG........ .......... .......... .......... [660]

MK257703 TTTTTTGTGT TTTCAACTTA GG........ .......... .......... .......... [660]

MK257704 TTCTTTGTGT TCTCAACTTA GG........ .......... .......... .......... [660]

#Md-v1FJ174253 TTCTTTGTGT TTTCAACTTA GG........ .......... .......... .......... [660]

#Md-v2FJ174254 TTTTTTGTGT TTTCAACTTA GG........ .......... .......... .......... [660]

#Md-v3FJ174255 TTCTTTGTGT TTTCAACTTA GG........ .......... .......... .......... [660]

#Md-v4FJ174256 TTCTTTGTGT TTTCAACTTA GG........ .......... .......... .......... [660]

#Md-v5FJ174257 TTCTTTGTGT TTTCAACTTA GG........ .......... .......... .......... [660]

#Md-v6FJ174258 TTCTTTGTGT TCTCAACTTA GG........ .......... .......... .......... [660]

#Md-v7FJ174259 TTCTTTGTGT TTTCAACTTA GG........ .......... .......... .......... [660]

#Md-v8FJ174260 TTCTTTGTGT TTTCAACTTA GG........ .......... .......... .......... [660]

#Md-v9FJ174261 TTCTTTGTGT TCTCAACTTA GG........ .......... .......... .......... [660]

#Md-v10FJ174262 TTTTTTGTGT TTTCAACTTA GG........ .......... .......... .......... [660]

#Md-v11FJ174263 TTTTTTGTGT TTTCAACTTA GG........ .......... .......... .......... [660]

#Md-v12FJ174264 TTCTTTGTGT TTTCAACTTA GG........ .......... .......... .......... [660]

#Md-v13FJ174265 TTCTTTGTGT TTTCAACTTA GG........ .......... .......... .......... [660]

#Md-v14FJ174266 TTCTTTGTGT TCTCAACTTA GG........ .......... .......... .......... [660]

#Md-v15FJ174267 TTCTTTGTGT TCTCAACTTA GG........ .......... .......... .......... [660]

1668

#AF281161aabys GATGCGAAAA CAATTTCTGT CCAACAATGG AACTCATATT CTGGAATTTT GAGTTTTCCC [720]

MK257685 .......... .......... .......... .....G.... .......... A....A.... [720]

MK257686 .......... .......... ...G...... .......... .......... A......... [720]

MK257687 .......... .......... .......... .....G.... .......... A....A.... [720]

MK257688 .......... .......... .......... .....G.... .......... A....A.... [720]

MK257689 .......... .......... .......... .....G.... .......... A....A.... [720]

MK257690 .......... .......... .......... .....G.... .......... A....A.... [720]

MK257691 .......... .......... .......... .....G.... .......... A....A.... [720]

MK257692 .......... .......... .......... .....G.... .......... A....A.... [720]

MK257693 .......... .......... .......... .......... .......... A....A.... [720]

MK257694 .......... .......... .......... .......... .......... A......... [720]

MK257695 .......... .......... .......... .......... .......... A....A.... [720]

MK257696 .......... .......... .......... .....G.... .......... A....A.... [720]

MK257697 .......... .......... .......... .....G.... .......... A....A.... [720]

MK257698 .......... .......... .......... .....G.... .......... A....A.... [720]

MK257699 .......... .......... .......... .......... .......... A......... [720]

MK257700 .......... .......... .......... .....G.... .......... A....A.... [720]

MK257701 .......... .......... .......... .....G.... .......... A....A.... [720]

MK257702 .......... .......... .......... .....G.... .......... A....A.... [720]

MK257703 .......... .......... .......... .....G.... .......... A....A.... [720]

MK257704 .......... .......... .......... .......... .......... A......... [720]

#Md-v1FJ174253 .......... .......... .......... .......... .......... .......... [720]

#Md-v2FJ174254 .......... .......... .......... .....G.... .......... A......... [720]

#Md-v3FJ174255 .......... .......... ...G...... .......... .......... A......... [720]

#Md-v4FJ174256 .......... .......... .......... .......... .......... .......... [720]

#Md-v5FJ174257 .......... .......... .......... .......... .......... .......... [720]

#Md-v6FJ174258 .......... .......... ...G...... .....G.... .......... A......... [720]

#Md-v7FJ174259 .......... .......... ...G...... .......... .......... A......... [720]

#Md-v8FJ174260 .......... .......... .......... .......... .......... A..C...... [720]

#Md-v9FJ174261 .......... .......... ...G...... .......... .......... A......... [720]

#Md-v10FJ174262 .......... .......... .......... .....G.... .......... A....A.... [720]

#Md-v11FJ174263 .......... .......... .......... .....G.... .......... A....A.... [720]

#Md-v12FJ174264 .......... .......... ...G...... .......... .......... .......... [720]

#Md-v13FJ174265 .......... .T........ .......... .......... .......... A......... [720]

#Md-v14FJ174266 .......... .......... .......... .......... .......... A....A.... [720]

#Md-v15FJ174267 .......... .......... .......... .....G.... .......... A....A.... [720]

#AF281161aabys TCGGCCCCAA CTATAGATGG AGCATTTTTG CCCGCAGATC CAATGACACT TTTGAAAACA [780]

MK257685 .......... .......... .......... .......... .......... G......... [780]

MK257686 .......... .......... .......... .......... .......... G......... [780]

MK257687 .......... .......... .......... .......... .......... G......... [780]

MK257688 .......... .......... .......... .......... .......... G......... [780]

MK257689 .......... .......... .......... .......... .......... G......... [780]

MK257690 .......... .......... .......... .......... .......... G......... [780]

MK257691 .......... .......... .......... ......A... .......... G......... [780]

MK257692 .......... .......... .......... .......... .......... G......... [780]

MK257693 .......... .......... .......... .......... .......... G......... [780]

MK257694 .......... .......... .......... .......... .......... G......... [780]

MK257695 .......... .......... .......... .......... .......... G......... [780]

MK257696 .......... .......... .......... .......... .......... G......... [780]

MK257697 .......... .......... .......... .......... .......... G......... [780]

MK257698 .......... .......... .......... .......... .......... G......... [780]

MK257699 .......... .......... .......... .......... .......... G......... [780]

MK257700 .......... .......... .......... .......... .......... G......... [780]

MK257701 .......... .......... .......... .......... .......... G......... [780]

MK257702 .......... .......... .......... .......... .......... G......... [780]

MK257703 .......... .......... .......... .......... .......... G......... [780]

MK257704 .......... .......... .......... .......... .......... G......... [780]

#Md-v1FJ174253 .......... .......... .......... ...------- ---------- ---------- [780]

#Md-v2FJ174254 .......... .......... .......... ...------- ---------- ---------- [780]

#Md-v3FJ174255 .......... .......... .......... ...------- ---------- ---------- [780]

#Md-v4FJ174256 .......... .......... .......... ...------- ---------- ---------- [780]

#Md-v5FJ174257 .......... .......... .......... ...------- ---------- ---------- [780]

#Md-v6FJ174258 .......... .......... .......... ...------- ---------- ---------- [780]

#Md-v7FJ174259 .......... .......... .......... ...------- ---------- ---------- [780]

#Md-v8FJ174260 .......... .......... .......... ...------- ---------- ---------- [780]

#Md-v9FJ174261 .......... .......... .......... ...------- ---------- ---------- [780]

#Md-v10FJ174262 .......... .......... .......... ...------- ---------- ---------- [780]

#Md-v11FJ174263 .......... .......... .......... ...------- ---------- ---------- [780]

#Md-v12FJ174264 .......... .......... .......... ...------- ---------- ---------- [780]

#Md-v13FJ174265 .......... .......... .......... ...------- ---------- ---------- [780]

#Md-v14FJ174266 .......... .......... .......... ...------- ---------- ---------- [780]

#Md-v15FJ174267 .......... .......... .......... ...------- ---------- ---------- [780]

#AF281161aabys GCAGATCTTA GCGGTTACGA TATTCTGATT GGAAATGTTA AAGATGA [827]

MK257685 .......... .......... .......... .......... ....... [827]

MK257686 .......... .......... .......... .......... ....... [827]

MK257687 .......... .......... .......... .......... ....... [827]

MK257688 .......... .......... .......... .......... ....... [827]

MK257689 .......... .......... .......... .......... ....... [827]

MK257690 .......... .......... .......... .......... ....... [827]

MK257691 .......... .......... .......... .......... ....... [827]

MK257692 .......... .......... .......... .......... ....... [827]

MK257693 .......... .......... .......... .......... ....... [827]

MK257694 .......... .......... .......... .......... ....... [827]

MK257695 .......... .......... .......... .......... ....... [827]

MK257696 .......... .......... .......... .......... ....... [827]

MK257697 .......... .......... .......... .......... ....... [827]

MK257698 .......... .......... .......... .......... ....... [827]

MK257699 .......... .......... .......... .......... ....... [827]

MK257700 .......... .......... .......... .......... ....... [827]

MK257701 .......... .......... .......... .......... ....... [827]

MK257702 .......... .......... .......... .......... ....... [827]

MK257703 .......... .......... .......... .......... ....... [827]

MK257704 .......... .......... .......... .......... ....... [827]

#Md-v1FJ174253 ---------- ---------- ---------- ---------- ------- [827]

#Md-v2FJ174254 ---------- ---------- ---------- ---------- ------- [827]

#Md-v3FJ174255 ---------- ---------- ---------- ---------- ------- [827]

#Md-v4FJ174256 ---------- ---------- ---------- ---------- ------- [827]

#Md-v5FJ174257 ---------- ---------- ---------- ---------- ------- [827]

#Md-v6FJ174258 ---------- ---------- ---------- ---------- ------- [827]

#Md-v7FJ174259 ---------- ---------- ---------- ---------- ------- [827]

#Md-v8FJ174260 ---------- ---------- ---------- ---------- ------- [827]

#Md-v9FJ174261 ---------- ---------- ---------- ---------- ------- [827]

#Md-v10FJ174262 ---------- ---------- ---------- ---------- ------- [827]

#Md-v11FJ174263 ---------- ---------- ---------- ---------- ------- [827]

#Md-v12FJ174264 ---------- ---------- ---------- ---------- ------- [827]

#Md-v13FJ174265 ---------- ---------- ---------- ---------- ------- [827]

#Md-v14FJ174266 ---------- ---------- ---------- ---------- ------- [827]

#Md-v15FJ174267 ---------- ---------- ---------- ---------- ------- [827]
